# Supplementary material for: “We must work tirelessly to promote …”: Mediating interpersonal commitment to material processes in English translation of Chinese political discourse
Source: PLoS One. 2025 Dec 12;20(12):e0338440. doi: 10.1371/journal.pone.0338440 (PMC12700425; doi:10.1371/journal.pone.0338440)
Supplement: Attachment — Submitted filename: review.doc [file pone.0338440.s002.doc]

This paper has investigated the translation choices in political texts from Chinese to English, focusing on the mediation of interpersonal commitment to circumstances coupled with material processes. Employing the theoretical framework of reinstantiation within Systemic Functional Linguistics (SFL), this research has provided a comprehensive description of how and why certain circumstances were shifted in translation, with reference to grammatical forms and possible motivations based on data extracted from a corpus of institutional translation.

The whole paper is divided into 7 parts. In Part One, the authors introduce the academic background of political translation. In Part Two, the authors review studies that have focused on political translation. In Part Three, the authors explain the theoretical framework of this paper. Part Four focuses on the methodology and presents the top ten verbs of material processes in Table One. Part Five, titled "Quantitative Findings," presents the results of this study. In this part, the authors not only present the research results but also analyze the translation methods. In Part Six, the authors discuss lexical choices in translation. In the end, the conclusion is drawn.

The presentation, writing skills, data and materials used, and the theoretical basis on which this paper relies are all rich and reliable. The data analysis is valuable and can be used.

This article has met the publication requirements.

Suggestions for Improvement:

It would be better if the authors provide several concrete examples in full sentences for Table 2 to prove the translation choices.

Examples from Ex. 2 lack concrete information, such as page numbers.
