# Supplementary material for: “We must work tirelessly to promote …”: Mediating interpersonal commitment to material processes in English translation of Chinese political discourse
Source: PLoS One. 2025 Dec 12;20(12):e0338440. doi: 10.1371/journal.pone.0338440 (PMC12700425; doi:10.1371/journal.pone.0338440)
Supplement: Attachment — Submitted filename: Response letter.docx [file pone.0338440.s003.docx]

Dear Professor Fasih Ahmed,

We have addressed all the issues raised in Journal Requirements and reviewer comments. However, please note that comments of Reviewer #2 seem to be irrelevant to our paper (PONE-D-25-02479) because it does not concern “digital resilience”. We therefore only responded to Reviewer #1 and Reviewer #3. Thank you very much!

Below are our responses marked in blue.

Journal Requirements:

When submitting your revision, we need you to address these additional requirements.

1.Please ensure that your manuscript meets PLOS ONE's style requirements, including those for file naming. The PLOS ONE style templates can be found at

https://journals.plos.org/plosone/s/file?id=wjVg/PLOSOne_formatting_sample_main_body.pdf and

https://journals.plos.org/plosone/s/file?id=ba62/PLOSOne_formatting_sample_title_authors_affiliations.pdf

//We have reformatted our manuscript to meet the style requirements.

2. Thank you for stating the following financial disclosure:

“Funder: Project of Guangdong Provincial Philosophy and Social Science Planning 2024

Project Title: A Study on the Projection Language Features in Political Discourse and Their Translation

Project Number: GD24CWY13”

Please state what role the funders took in the study. If the funders had no role, please state: "The funders had no role in study design, data collection and analysis, decision to publish, or preparation of the manuscript."

If this statement is not correct you must amend it as needed.

Please include this amended Role of Funder statement in your cover letter; we will change the online submission form on your behalf.

// We have added "The funders had no role in study design, data collection and analysis, decision to publish, or preparation of the manuscript." in the cover letter and manuscript. Thank you.

3. We note that your Data Availability Statement is currently as follows: All relevant data are within the manuscript and in Supporting Information files.

Please confirm at this time whether or not your submission contains all raw data required to replicate the results of your study. Authors must share the “minimal data set” for their submission. PLOS defines the minimal data set to consist of the data required to replicate all study findings reported in the article, as well as related metadata and methods (https://journals.plos.org/plosone/s/data-availability#loc-minimal-data-set-definition).

For example, authors should submit the following data:

- The values behind the means, standard deviations and other measures reported;

- The values used to build graphs;

- The points extracted from images for analysis.

Authors do not need to submit their entire data set if only a portion of the data was used in the reported study.

If your submission does not contain these data, please either upload them as Supporting Information files or deposit them to a stable, public repository and provide us with the relevant URLs, DOIs, or accession numbers. For a list of recommended repositories, please see https://journals.plos.org/plosone/s/recommended-repositories.

If there are ethical or legal restrictions on sharing a de-identified data set, please explain them in detail (e.g., data contain potentially sensitive information, data are owned by a third-party organization, etc.) and who has imposed them (e.g., an ethics committee). Please also provide contact information for a data access committee, ethics committee, or other institutional body to which data requests may be sent. If data are owned by a third party, please indicate how others may request data access.

// We have provided a link to the data of our paper as below.

<https://www.researchgate.net/publication/391812264_xijinpingtanzhiguolizhengyuliaobiao_advno_adv_20240315_LJC_20240317>

We also added the following statement in the article

**Data:** The data of this study can be accessed through this link: https://www.researchgate.net/publication/391812264_xijinpingtanzhiguolizhengyuliaobiao_advno_adv_20240315_LJC_20240317

4. Please ensure that you refer to Figure 1 in your text as, if accepted, production will need this reference to link the reader to the figure.

// Thank you. We have referred to Figure 1 as follows:

We also refer to the Appraisal Theory (Martin & White 2005) to examine the interpersonal meaning in translation. In particular, we considered the system of graduation, as displayed in Figure 1. Graduation is one of the three main systems of meanings used to express positive or negative evaluations in discourse,. Graduation deals with adjusting the strength or intensity of an evaluative stance. It operates along two axes: force and focus.

Reviewer #1: This paper examines the ways in which interpersonal commitment in the English translation of Chinese political discourse is mediated through evaluative items within appraisal system framework. While the topic is interesting and the method is proper, there are a couple of areas where the paper needs to be reworked in order to be publishable.

1. The authors need to provide more justification of corpus selection. While the corpus is authoritative, further discussion on potential biases or limitations in using Xi Jinping’s discourse as the primary dataset would increase the generalization of the research results.

//Thank you. We have added the limitations of the data as follows

Despite its strengths, the corpus has limitations. First, the corpus is limited to Xi Jinping’s discourse, excluding other political figures or texts, which may restrict the generalizability of findings to broader Chinese political discourse. Second, manual annotation of grammatical forms and interpersonal commitment risks interpretive bias.

2. The authors need to be aware of the potential international readers, not just Chinese readers. So when, for example, writing sth like “president Xi”, it might be more acceptable by using “Chinese president Xi Jinping” when it firstly is used.

//Thanks. We have replaced president Xi with Chinese president Xi Jinping

3. Some sure it is reliable to make a certain argument. For example, “which differs from previous findings on mental processes”, here it would be helpful if some references are included.

// We have added the reference: Chen et al. 2023

4. Some sections contain very complex sentence structures. Please break them down, which is also related to comment 2 to take readers into account when writing English.

//Thank you. The complexity of some sentences might be due to theoretical thickness of the research design. We have tried to break down a few long sentences.

Basically, I would recommend it for publication after minor revision.

Reviewer #3: This paper has investigated the translation choices in political texts from Chinese to English, focusing on the mediation of interpersonal commitment to circumstances coupled with material processes. Employing the theoretical framework of reinstantiation within Systemic Functional Linguistics (SFL), this research has provided a comprehensive description of how and why certain circumstances were shifted in translation, with reference to grammatical forms and possible motivations based on data extracted from a corpus of institutional translation.

The whole paper is divided into 7 parts. In Part One, the authors introduce the academic background of political translation. In Part Two, the authors review studies that have focused on political translation. In Part Three, the authors explain the theoretical framework of this paper. Part Four focuses on the methodology and presents the top ten verbs of material processes in Table One. Part Five, titled "Quantitative Findings," presents the results of this study. In this part, the authors not only present the research results but also analyze the translation methods. In Part Six, the authors discuss lexical choices in translation. In the end, the conclusion is drawn.

The presentation, writing skills, data and materials used, and the theoretical basis on which this paper relies are all rich and reliable. The data analysis is valuable and can be used.

This article has met the publication requirements.

Suggestions for Improvement:

It would be better if the authors provide several concrete examples in full sentences for Table 2 to prove the translation choices.

// Thank you for your suggestion. Space does not allow us to provide full sentence examples. However, we offered a snapshot of our excel annotations to prove the translation choices. See Figure 2.

Examples from Ex. 2 lack concrete information, such as page numbers.

// Thanks. All examples are extracted from a corpus. So there is no information about page numbers.

Thank you very much for your helpful comments!
