# Supplementary material for: “We must work tirelessly to promote …”: Mediating interpersonal commitment to material processes in English translation of Chinese political discourse
Source: PLoS One. 2025 Dec 12;20(12):e0338440. doi: 10.1371/journal.pone.0338440 (PMC12700425; doi:10.1371/journal.pone.0338440)
Supplement: Attachment — Submitted filename: PONE-D-25-02479_Reviewers report.docx [file pone.0338440.s005.docx]

This manuscript touches on a very interesting topic. The selected political texts provide a good source for investigating the shifts of interpersonal meaning between the Chinese original text (ST) and its English translated text (TT). SFL is a systemic approach to analyse how interpersonal meaning is instantiated in the ST and how the meaning is re-instantiated in the TT. The methodology shows that, by focusing on the pattern of “circumstances + material processes”, the authors had made a scrutinized examination of the translation shifts in grammatical forms. The findings provide a good foundation for further investigation into the potential translation shifts in terms of interpersonal commitment. Therefore, the overall design of this project is quite promising.

The manuscript is full of potential. However, a few issues need to be addressed before publication.

1. Page 5, *“Prescriptive studies offer detailed description of what really happened in political*

*translation, mostly relying on a corpus method.”*

There is a typo; this should be an explanation of ‘Descriptive studies’. Furthermore, after introducing the prescriptive and descriptive approaches, it is better to clarify which approach this project takes.

2. Page 5-6, *“More specifically, the instantiation cline involves system, genre/register, text type, text, and reading, where system represents the most general end of meaning potential and reading stands for subjectified meaning, the most actual end.”*

This may be a bit hard to follow for readers from a non-SFL background. Ideally, it is better to break it down a bit for the readers. The authors may consider, at least, providing a figure of the instantiation cline and directing the readers to a relevant reference. The authors can use the one in Martin and White (2005) or another work they prefer.

3. Page 11, *“The top five circumstances are buduan 不断 (continually), quanmian 全面 (fully), jiji 积极 (actively), xietiao 协调 (coordinately), and qieshi 切实 (practically)…These circumstances boost the force of the material processes, thus raising the mobilizing effects of the political communication.”*

The manuscript states that all circumstances included in this project refer to an instance of intensification. From the perspective of Appraisal, it seems hard to take *协调 (coordinately)* and *qieshi 切实 (practically)* as instances of intensification. Would the authors please clarify the reasons for identifying the two items as ‘GRADUATION: intensification’?

3. Page 14, Example (4), under the heading of *“Using prepositional phrases to achieve equal interpersonal commitment”*:

*[ST]*

*以 更加 开放 的 胸襟 和 更加 积极 的 态度 [[促进]] 地区 合作 .*

*with an opener mind and more positive attitude promote regional cooperation*

*[TT]*

*… and promoting regional cooperation with an open mind and enthusiasm.*

Comparatives are commonly used as intensifiers; they usually indicate an upscaling of intensity or of quantity. In this example, the two comparatives in the ST were omitted in the TT, this would have an impact on the rhetorical force and eventually affect the interpersonal commitment. Would the authors please clarify the reason why this example was taken as achieving an equal interpersonal commitment?

4. Page 15, Example (8):

*[ST]*

*不久 将 全面 [[建成]] 小康社会,*

*soon will comprehensively build a moderately prosperous society.*

*[TT]*

*… and will soon bring the building of a moderately prosperous society to a successful completion.*

*“…In example (8), the degree adverb “quanmian 全面 fully” was changed into the adjective ‘successful’, indicating a judgment of capacity.”*

In the English text – ‘*a successful completion*’ – *completion* as the target of this evaluation refers to a thing rather than a behaviour.

5. Page 17, Example (10):

*[ST]*

*我们 要 坚持不懈 [[抓好]] 宪法 实施 工作 …*

*We must persistently without slack grasp the Constitution implementation work*

*[TT]*

*We must persistently ensure the implementation of the Constitution … Idiom phrase*

*“…In example (10), jianchi buxie 坚持不懈 is turned into “persistently”, whereby the rich*

*connotation of determination of the phrase is lost in translation.”*

The authors will need to elaborate on the explanation for this instance, because at the semantic level there seems to be no shift.

6. Page 19, Example (13):

*[ST]*

*… 稳步 [[推进]] 城镇化 健康 发展*

*steadily promote urbanization’s healthy development*

*[TT]*

*… and promote well-planned and healthy urbanization.*

*“In example (13), the adverb ‘wenbu 稳步 (steadily)’ is converted to an adjective ‘well-planned’, which serves as a modifier of the head ‘urbanization’. Using terms from appraisal theory, the meaning of force has been turned into appreciation of ‘composition’ [39], that is, attaching the value of balance to ‘urbanization’.”*

Would the authors please clarify the reason for identifying *wenbu 稳步 (steadily)* as an instance of ‘GRADUATION: intensification’?
