# Supplementary material for: “We must work tirelessly to promote …”: Mediating interpersonal commitment to material processes in English translation of Chinese political discourse
Source: PLoS One. 2025 Dec 12;20(12):e0338440. doi: 10.1371/journal.pone.0338440 (PMC12700425; doi:10.1371/journal.pone.0338440)
Supplement: Attachment — Submitted filename: Response_letter_auresp_3.docx [file pone.0338440.s007.docx]

Dear Editor,

We are grateful for the helpful comment of the reviewer. We have addressed each issue raised by the reviewer. Please see below for our response.

This manuscript touches on a very interesting topic. The selected political texts provide a good source for investigating the shifts of interpersonal meaning between the Chinese original text (ST) and its English translated text (TT). SFL is a systemic approach to analyse how interpersonal meaning is instantiated in the ST and how the meaning is re-instantiated in the TT. The methodology shows that, by focusing on the pattern of “circumstances + material processes”, the authors had made a scrutinized examination of the translation shifts in grammatical forms. The findings provide a good foundation for further investigation into the potential translation shifts in terms of interpersonal commitment. Therefore, the overall design of this project is quite promising.

The manuscript is full of potential. However, a few issues need to be addressed before publication.

1. Page 5, *“Prescriptive studies offer detailed description of what really happened in political*

*translation, mostly relying on a corpus method.”*

There is a typo; this should be an explanation of ‘Descriptive studies’. Furthermore, after introducing the prescriptive and descriptive approaches, it is better to clarify which approach this project takes.

//Response: Thank you for pointing that out. We have corrected the typo and clarify the approach of this study as follows:

Descriptive studies offer detailed description of what really happened in political translation, mostly relying on a corpus method. The data generated out of a large-scale corpus provide insights into linguistic features of various political texts and patterns of translation choices [26-28]. Translation studies of interpersonal resources have been particularly fruitful in this respect [7, 8, 11, 12, 13, 14]. Yu and Wang [7] find that in Chinese-to-English translation, high-valued modal expressions in the source texts are often omitted or translated into lower-valued expressions, while median-valued modal expressions tend to be retained. Additionally, the translated texts exhibit a shift towards a more target-oriented tone aligned with English communicative conventions and the intended persuasive function in the target culture. This study adopts exactly this descriptive approach to examine interpersonal shifts in political translation.

2. Page 5-6, *“More specifically, the instantiation cline involves system, genre/register, text type, text, and reading, where system represents the most general end of meaning potential and reading stands for subjectified meaning, the most actual end.”*

This may be a bit hard to follow for readers from a non-SFL background. Ideally, it is better to break it down a bit for the readers. The authors may consider, at least, providing a figure of the instantiation cline and directing the readers to a relevant reference. The authors can use the one in Martin and White (2005) or another work they prefer.

//Response: Thank you. We have added the following figure to illustrate the instantiation cline.

**Figure 1. Instantiation as a cline from reading to system (Martin [30])**

3. Page 11, *“The top five circumstances are buduan 不断 (continually), quanmian 全面 (fully), jiji 积极 (actively), xietiao 协调 (coordinately), and qieshi 切实 (practically)…These circumstances boost the force of the material processes, thus raising the mobilizing effects of the political communication.”*

The manuscript states that all circumstances included in this project refer to an instance of intensification. From the perspective of Appraisal, it seems hard to take *协调 (coordinately)* and *qieshi 切实 (practically)* as instances of intensification. Would the authors please clarify the reasons for identifying the two items as ‘GRADUATION: intensification’?

//Response: Thank you. This is indeed a good question. Actually, we included circumstances with evaluative sense of frequency, degree and manner, going beyond the scope of ‘GRADUATION: intensification’. We have corrected the relevant parts to avoid confusion as follows:

In the case of the current study, we aim to observe how the meaning of force has come to enhance the material processes in Chinese political texts, and the correspondent translation choices. That means, only those circumstances that carry the meaning of evaluative force (i.e. degree, frequency and manner) are included for the current study.

3. Page 14, Example (4), under the heading of *“Using prepositional phrases to achieve equal interpersonal commitment”*:

*[ST]*

*以 更加 开放 的 胸襟 和 更加 积极 的 态度 [[促进]] 地区 合作 .*

*with an opener mind and more positive attitude promote regional cooperation*

*[TT]*

*… and promoting regional cooperation with an open mind and enthusiasm.*

Comparatives are commonly used as intensifiers; they usually indicate an upscaling of intensity or of quantity. In this example, the two comparatives in the ST were omitted in the TT, this would have an impact on the rhetorical force and eventually affect the interpersonal commitment. Would the authors please clarify the reason why this example was taken as achieving an equal interpersonal commitment?

//Response:

Thank you. This is a mistake. We checked our annotation in our dataset. This is indeed a case of evaluative downgrade. So we changed the example as follows:

4. Page 15, Example (8):

*[ST]*

*不久 将 全面 [[建成]] 小康社会,*

*soon will comprehensively build a moderately prosperous society.*

*[TT]*

*… and will soon bring the building of a moderately prosperous society to a successful completion.*

*“…In example (8), the degree adverb “quanmian 全面 fully” was changed into the adjective ‘successful’, indicating a judgment of capacity.”*

In the English text – ‘*a successful completion*’ – *completion* as the target of this evaluation refers to a thing rather than a behaviour.

//Response:

This is a case of grammatical metaphor. Completion, while it is a thing, is actually a nominalization of a verb, referring to the action of “complete”, hence a judgment of how well the thing is done.

5. Page 17, Example (10):

*[ST]*

*我们 要 坚持不懈 [[抓好]] 宪法 实施 工作 …*

*We must persistently without slack grasp the Constitution implementation work*

*[TT]*

*We must persistently ensure the implementation of the Constitution … Idiom phrase*

*“…In example (10), jianchi buxie 坚持不懈 is turned into “persistently”, whereby the rich*

*connotation of determination of the phrase is lost in translation.”*

The authors will need to elaborate on the explanation for this instance, because at the semantic level there seems to be no shift.

//Response: Thank you. We explained this as follows:

The interpersonal commitment is weakened because the translation reduces the emphatic double-structure "坚持 (persist) + 不懈 (without slack)" into the single word "persistently." This loss of the reinforcing element "不懈" diminishes the original sense of unwavering, tenacious determination.

6. Page 19, Example (13):

*[ST]*

*… 稳步 [[推进]] 城镇化 健康 发展*

*steadily promote urbanization’s healthy development*

*[TT]*

*… and promote well-planned and healthy urbanization.*

*“In example (13), the adverb ‘wenbu 稳步 (steadily)’ is converted to an adjective ‘well-planned’, which serves as a modifier of the head ‘urbanization’. Using terms from appraisal theory, the meaning of force has been turned into appreciation of ‘composition’ [39], that is, attaching the value of balance to ‘urbanization’.”*

Would the authors please clarify the reason for identifying *wenbu 稳步 (steadily)* as an instance of ‘GRADUATION: intensification’?

//Response:

wenbu 稳步 (steadily) is a case of manner, not intensification. See our correction above. Thank you!

Many thanks again for the correction and insightful comment.
