# Supplementary material for: “We must work tirelessly to promote …”: Mediating interpersonal commitment to material processes in English translation of Chinese political discourse
Source: PLoS One. 2025 Dec 12;20(12):e0338440. doi: 10.1371/journal.pone.0338440 (PMC12700425; doi:10.1371/journal.pone.0338440)
Supplement: Attachment — Submitted filename: PONE-D-25-02479_Reviewers report2.docx [file pone.0338440.s008.docx]

Page 15, Example (8):

[ST]

不久 将 全面 [[建成]] 小康社会,

soon will comprehensively build a moderately prosperous society.

[TT]

… and will soon bring the building of a moderately prosperous society to a successful completion.

“…In example (8), the degree adverb “quanmian 全面 fully” was changed into the

adjective ‘successful’, indicating a judgment of capacity.”

In the English text – ‘a successful completion’ – completion as the target of this

evaluation refers to a thing rather than a behaviour.

//Response:

This is a case of grammatical metaphor. Completion, while it is a thing, is actually a nominalization of a verb, referring to the action of “complete”, hence a judgment of how

well the thing is done.

- Yes, we can discuss this case as inscribed appreciation & invoked judgement. The authors may consider providing the above clarification for the reader.

Page 11, “The top five circumstances are buduan 不断 (continually), quanmian 全面 (fully), jiji 积极 (actively), xietiao 协调 (coordinately), and qieshi 切实 (practically)…These circumstances boost the force of the material processes, thus raising the mobilizing effects of the political communication.” The manuscript states that all circumstances included in this project refer to an instance of intensification. From the perspective of Appraisal, it seems hard to take 协调 (coordinately) and qieshi 切实 (practically) as instances of intensification. Would the authors please clarify the reasons for identifying the two items as ‘GRADUATION:

intensification’?

//Response: Thank you. This is indeed a good question. Actually, we included circumstances with evaluative sense of frequency, degree and manner, going beyond the scope of ‘GRADUATION: intensification’. We have corrected the relevant parts to avoid confusion as follows:

In the case of the current study, we aim to observe how the meaning of force has come to enhance the material processes in Chinese political texts, and the correspondent translation choices. That means, only those circumstances that carry the meaning of evaluative force (i.e. degree, frequency and manner) are included for the current study.

- The term ‘intensification’ in the GRADUATION system may cause some confusion. It refers to the author’s up/down scaling of qualities and processes, and manner is an important resource. For this case, the attitudinal meanings in 协调 (coordinately) and 切实 (practically) are quite obvious. However, my concern is whether these two items indicate a subjective scaling.

Page 19, Example (13):

[ST]

… 稳步 [[推进]] 城镇化 健康 发展

steadily promote urbanization’s healthy development

[TT]

… and promote well-planned and healthy urbanization.

“In example (13), the adverb ‘wenbu 稳步 (steadily)’ is converted to an adjective ‘wellplanned’,

which serves as a modifier of the head ‘urbanization’. Using terms from appraisal theory, the meaning of force has been turned into appreciation of ‘composition’ [39], that is, attaching the value of balance to ‘urbanization’.” Would the authors please clarify the reason for identifying wenbu 稳步 (steadily) as an instance of ‘GRADUATION: intensification’?

//Response:

wenbu 稳步 (steadily) is a case of manner, not intensification. See our correction above.

- The same concern as above.
