# Supplementary material for: “We must work tirelessly to promote …”: Mediating interpersonal commitment to material processes in English translation of Chinese political discourse
Source: PLoS One. 2025 Dec 12;20(12):e0338440. doi: 10.1371/journal.pone.0338440 (PMC12700425; doi:10.1371/journal.pone.0338440)
Supplement: Attachment — Submitted filename: Response_letter_auresp_4.docx [file pone.0338440.s009.docx]

Dear Editor,

We are grateful for your meticulous work. Please see below for our response.

If the reviewer comments include a recommendation to cite specific previously published works, please review and evaluate these publications to determine whether they are relevant and should be cited. There is no requirement to cite these works unless the editor has indicated otherwise.

//Response:

One reviewer recommended to cite a previously published work to indicate how findings of the current study differed from previous ones (Chen et al. 2023). We have evaluated it and confirmed that it is necessary.

Please review your reference list to ensure that it is complete and correct. If you have cited papers that have been retracted, please include the rationale for doing so in the manuscript text, or remove these references and replace them with relevant current references. Any changes to the reference list should be mentioned in the rebuttal letter that accompanies your revised manuscript. If you need to cite a retracted article, indicate the article’s retracted status in the References list and also include a citation and full reference for the retraction notice.

//Response:

We have reviewed the reference list and confirmed that it is complete and correct.

Additional Editor Comments (if provided):

Some minor revisions as suggested by Reviewer 1 still need to be responded to and integrated into the paper itself.

Reviewer #4: Page 15, Example (8):

[ST]

不久 将 全面 [[建成]] 小康社会,

soon will comprehensively build a moderately prosperous society.

[TT]

… and will soon bring the building of a moderately prosperous society to a successful completion.

“…In example (8), the degree adverb “quanmian 全面 fully” was changed into the

adjective ‘successful’, indicating a judgment of capacity.”

In the English text – ‘a successful completion’ – completion as the target of this

evaluation refers to a thing rather than a behaviour.

//Response:

This is a case of grammatical metaphor. Completion, while it is a thing, is actually a nominalization of a verb, referring to the action of “complete”, hence a judgment of how well the thing is done.

- Yes, we can discuss this case as inscribed appreciation & invoked judgement. The authors may consider providing the above clarification for the reader.

//Response:

Thank you. It is indeed more accurate to use inscribed appreciation & invoked judgement to describe the phenomenon. We have added the discussion in the article as follows

More specifically, it is a case of inscribed appreciation and invoked judgement. “Completion”, while it is a thing for the target of appreciation, is actually a nominalization of a verb, referring to the action of “complete”, hence a judgment of how well the thing is done.

Page 11, “The top five circumstances are buduan 不断 (continually), quanmian 全面 (fully), jiji 积极 (actively), xietiao 协调 (coordinately), and qieshi 切实 (practically)…These circumstances boost the force of the material processes, thus raising the mobilizing effects of the political communication.” The manuscript states that all circumstances included in this project refer to an instance of intensification. From the perspective of Appraisal, it seems hard to take 协调 (coordinately) and qieshi 切实 (practically) as instances of intensification. Would the authors please clarify the reasons for identifying the two items as ‘GRADUATION:

intensification’?

//Response: Thank you. This is indeed a good question. Actually, we included circumstances with evaluative sense of frequency, degree and manner, going beyond the scope of ‘GRADUATION: intensification’. We have corrected the relevant parts to avoid confusion as follows:

In the case of the current study, we aim to observe how the meaning of force has come to enhance the material processes in Chinese political texts, and the correspondent translation choices. That means, only those circumstances that carry the meaning of evaluative force (i.e. degree, frequency and manner) are included for the current study.

- The term ‘intensification’ in the GRADUATION system may cause some confusion. It refers to the author’s up/down scaling of qualities and processes, and manner is an important resource. For this case, the attitudinal meanings in 协调 (coordinately) and 切实 (practically) are quite obvious. However, my concern is whether these two items indicate a subjective scaling.

//Response:

You are right to point out that 协调 (coordinately) and 切实 (practically) do not necessarily intensify the verb in terms of degree. However, our research design deliberately sets out to track any evaluative adverb—regardless of whether it inherently scales the process up or down—as long as it carries attitudinal meaning. In other words, some of the adverbs involve scaling of the process, some might not. But we counted all of them as long as they have evaluative meanings. And we wanted to observe how these evaluative meanings are upscaled or downscaled in translation in terms of interpersonal commitment.

The term ‘intensification’ in the GRADUATION system may cause some confusion.

//Response:

The term ‘intensification’ was replaced by ‘evaluative force’ to avoid confusion.

Page 19, Example (13):

[ST]

… 稳步 [[推进]] 城镇化 健康 发展

steadily promote urbanization’s healthy development

[TT]

… and promote well-planned and healthy urbanization.

“In example (13), the adverb ‘wenbu 稳步 (steadily)’ is converted to an adjective ‘wellplanned’,

which serves as a modifier of the head ‘urbanization’. Using terms from appraisal theory, the meaning of force has been turned into appreciation of ‘composition’ [39], that is, attaching the value of balance to ‘urbanization’.” Would the authors please clarify the reason for identifying wenbu 稳步 (steadily) as an instance of ‘GRADUATION: intensification’?

//Response:

wenbu 稳步 (steadily) is a case of manner, not intensification. See our correction above.

- The same concern as above.

//Response:

Thank you for the concern. Whether wenbu 稳步 (steadily) involves subjective scaling does not impact our findings because this study aims to observe the translation shifts of the adverbs containing evaluative meanings. 稳步 (steadily) has a meaning of “steady” and “moderate”. That is why we included this item for analysis for translation shifts.

Many thanks again for Reviewer’s comments and discussion. They are helpful for improvement of the paper.
