# Supplementary material for: “We must work tirelessly to promote …”: Mediating interpersonal commitment to material processes in English translation of Chinese political discourse
Source: PLoS One. 2025 Dec 12;20(12):e0338440. doi: 10.1371/journal.pone.0338440 (PMC12700425; doi:10.1371/journal.pone.0338440)
Supplement: Attachment — Submitted filename: Response_letter_auresp_5.docx [file pone.0338440.s010.docx]

Dear Editor,

We are grateful for your meticulous work. Please see below for our response.

Reviewer #4: Page 11, “The top five circumstances are buduan 不断 (continually), quanmian 全面 (fully), jiji 积极 (actively), xietiao 协调 (coordinately), and qieshi 切实(practically)…These circumstances boost the force of the material processes, thus raising the mobilizing effects of the political communication.” The manuscript states that all circumstances included in this project refer to an instance of intensification.

From the perspective of Appraisal, it seems hard to take 协调 (coordinately) and qieshi切实 (practically) as instances of intensification. Would the authors please clarify the reasons for identifying the two items as ‘GRADUATION:intensification’?

//Response: Thank you. This is indeed a good question. Actually, we included circumstances with evaluative sense of frequency, degree and manner, going beyond the scope of ‘GRADUATION: intensification’. We have corrected the relevant parts to avoid confusion as follows:

In the case of the current study, we aim to observe how the meaning of force has come to enhance the material processes in Chinese political texts, and the correspondent translation choices. That means, only those circumstances that carry the meaning of evaluative force (i.e. degree, frequency and manner) are included for the current study.

- The term ‘intensification’ in the GRADUATION system may cause some confusion. It refers to the author’s up/down scaling of qualities and processes, and manner is an important resource. For this case, the attitudinal meanings in 协调 (coordinately) and切实 (practically) are quite obvious. However, my concern is whether these two items indicate a subjective scaling.

//Response:

You are right to point out that 协调 (coordinately) and 切实 (practically) do not necessarily intensify the verb in terms of degree. However, our research design deliberately sets out to track any evaluative adverb—regardless of whether it inherently scales the process up or down—as long as it carries attitudinal meaning. In other words, some of the adverbs involve scaling of the process, some might not. But we counted all of them as long as they have evaluative meanings. And we wanted to observe how these evaluative meanings are upscaled or downscaled in translation in terms of interpersonal commitment.

- Thanks for the clarification. I would recommend the authors clarify in the ‘theoretical framework’ that the analyses of this paper were concerned with GRADUATION and ATTITUDE. This will be my last comment. I have no further concerns.

//Response:

Thank you. We have added a few words and a paragraph to clarify our research concerns as follows:

We also refer to the Appraisal Theory [39] to examine the interpersonal meaning in translation. In particular, we considered the system of graduation and attitude, as displayed in Fig 2. Graduation is one of the three main systems of meanings used to express positive or negative evaluations in discourse. Graduation deals with adjusting the strength or intensity of an evaluative stance. It operates along two axes: force and focus.

Force concerns the intensity or amount of the evaluated phenomenon. Force can be either "raised" (upscaled) or "lowered" (downscaled). For example, the words "very," "extremely," or "somewhat" can be used to modify the force of an adjective or adverb.

Focus sharpens or blurs the boundaries of the evaluative term. Under focus, we can distinguish between "sharpening" (specifying a term more precisely) and "softening" (vagueness or imprecision). For instance, words like "real" or "true" can sharpen the focus of a category, while "sort of" or "kind of" can soften it.

The Attitude subsystem represents choices of expressing feelings and evaluations. It is divided into three core domains: Affect, which deals with emotional responses and feelings; Judgement, which concerns the ethical assessment of behavior and character; and Appreciation, which evaluates the aesthetic and social value of objects, texts, and phenomena.

In the case of the current study, we aim to observe how the meaning of force and attitude has come to enhance the material processes in Chinese political texts, and the correspondent translation choices. That means, only those circumstances that carry the meaning of evaluative force (i.e. degree, frequency and manner) are included for the current study.

Many thanks again for Reviewer’s comments and discussion. They are helpful for improvement of the paper.
